# LC-MS-based conventional metabolomics combined with machine learning models to identify metabolic markers for the diagnosis of type I diabetes

**DOI:** 10.3389/fendo.2025.1588718

**Published:** 2025-08-07

**Authors:** Muhadasi Tuerxunyiming, Qing Zhao, Qiaosheng Hu, Ping Zhu, Shiting Zhu

**Affiliations:** ^1^ Zhejiang University School of Medicine, Hangzhou, China; ^2^ School of Medicine, Hangzhou City University, Hangzhou, China; ^3^ Endocrinology Department, Lianshui County People’s Hospital, Huai’an, Jiangsu, China; ^4^ Endocrinology Department, The Affiliated Chuzhou Hospital of Traditional Chinese Medicine of Jiangsu College of Nursing, Huai’an, Jiangsu, China; ^5^ Rehabilitation Medicine Department, Lianshui County People’s Hospital, Huai’an, Jiangsu, China

**Keywords:** type 1 diabetes, metabolomics, LC-MS, metabolic markers, LASSO

## Abstract

**Background:**

Changes in certain metabolites are linked to an increased risk of type I diabetes (T1D), making metabolite analysis a valuable tool for T1D diagnosis and treatment. This study aimed to identify a metabolic signature linked with T1D.

**Methods:**

Untargeted metabolomic profiling was performed using liquid chromatography–mass spectrometry (LC-MS) on peripheral blood samples from T1D patients (n = 45) and healthy controls (n = 40). Data preprocessing and quality control were conducted using MetaboAnalyst 4.0. Differential metabolites (DMs) were identified via Wilcoxon rank-sum test (P< 0.05), and key diagnostic markers were selected using least absolute shrinkage and selection operator (LASSO) regression. A streptozotocin (STZ)-induced diabetic rat model was used for *in vivo* validation.

**Results:**

A total of 157 annotated metabolites were detected (58 in ESI− and 99 in ESI+ mode). Twenty-six DMs were identified, including 25 upregulated and 1 downregulated in the T1D group, mainly involving Acylcarnitines and xanthine metabolites. LASSO regression selected Hydroxyhexadecanoyl carnitine, Propionylcarnitine, and Valerylcarnitine as candidate markers. In the rat model, Hydroxyhexadecanoyl carnitine and Valerylcarnitine demonstrated strong diagnostic performance, with AUCs of 0.9383 (95% CI: 0.8786–0.9980) and 0.8395 (95% CI: 0.7451–0.9338), respectively (P< 0.01).

**Conclusion:**

Hydroxyhexadecanoyl carnitine and Valerylcarnitine are closely linked to altered lipid oxidation in T1D and show strong potential as diagnostic biomarkers. These findings provide new insights into the metabolic basis of T1D and offer promising targets for early detection.

## Introduction

Type I diabetes (T1D) is a chronic autoimmune disease characterized by the destruction of insulin-producing cells in the pancreas, leading to insufficient insulin production and the inability to regulate blood glucose levels properly (1, 2). T1D primarily affects children and adolescents, significantly impacting their quality of life and life expectancy. Globally, an estimated 1.9 million children under the age of 15 are affected by T1D, with approximately 77,000 new cases diagnosed each year, representing an annual increase of about 3% (3). Although the exact causes of T1D remain unclear, it is believed to result from a combination of genetic, environmental, infectious, and nutritional factors (4). Research has indicated that specific changes in metabolites may be linked to an elevated risk of developing T1D (5–7). Metabolite analysis has thus emerged as a valuable tool in both the diagnosis and treatment of T1D, prompting extensive efforts to clarify the relationship between T1D and metabolic changes.

Metabolomics is the study of small molecules produced by metabolic reactions within biological systems. Low molecular weight molecules found in human body fluids, such as blood or urine, can be measured using analytical chemistry techniques, including gas or liquid chromatography coupled with mass spectrometry (8–11). This approach is instrumental in studying disease-related metabolic markers and predicting diseases in clinical settings. For example, Liu et al. found that metabolites like triglycerides, amino acids, and small intermediate compounds were associated with type 2 diabetes (T2D) and could serve as predictors of future T2D onset (12). Similarly, Takayuki Teruya and colleagues identified 33 metabolic markers, 12 of which were enriched in erythrocytes, that were linked to dementia, confirming their association with disease progression (13). In obesity research, metabolomic analyses highlighted significant differences in gamma-glutamyl, branched-chain amino acid metabolism, and triacylglycerols in lipid profiles of obese patients (10). These examples demonstrate the value of metabolomics in uncovering disease-specific metabolic markers.

In this study, to explore the metabolic marker profiles of T1D, we conducted routine metabolomics analysis using liquid chromatography–mass spectrometry (LC-MS) technology. Peripheral blood samples were collected from pediatric T1D patients and healthy controls, and a machine learning model was applied to identify key metabolic markers.

## Materials and methods

### Objects

In this study, 45 pediatric patients diagnosed with T1D at Hangzhou City University were included, along with 40 healthy non-diabetic controls hospitalized for other reasons. The inclusion criteria for T1D participants were based on the 2020 American Diabetes Association (ADA) diagnostic guidelines, including fasting plasma glucose (FPG) ≥ 7.0 mmol/L and/or glycated hemoglobin (HbA1c) ≥ 6.5%, in combination with positive pancreatic autoantibodies (e.g., GADA, ICA, or IAA) and insulin dependence. Healthy controls were defined as individuals with FPG< 5.6 mmol/L, HbA1c< 5.7%, and no known history of diabetes, metabolic disorders, or autoimmune disease. The case and control groups were matched for age and sex. Inclusion criteria were based on the new diagnostic guidelines from the Diabetes Society of the Chinese Medical Association. Diagnosis required (a) plus two or more of the following: (b) ketonuria, (c) nocturia >3–4 times per night, (d) significant weight loss, (e) a first-degree relative with diabetes requiring insulin, or (f) a history of autoimmune disease. Patients with diabetic ketoacidosis were excluded. Peripheral blood samples were collected from both groups and stored at -80°C. Informed consent was obtained from all participants, and the study was approved by the Ethics Committee of Hangzhou City University.

Sample metabolites were extracted by mixing 25 μL of plasma with 300 μL of cold acetonitrile:methanol (5:4:1, v:v:v) and vortexing the mixture. The sample was then placed on ice for 30 minutes before being centrifuged at 12,000 rpm for 10 minutes at 4°C. The supernatant was collected and transferred to a suitable LC/MS vial for analysis.

### LC-MS technology for metabolomic analysis

Metabolomic analysis of peripheral blood samples was conducted using LC-MS technology. Data were collected with a 4000 QTRAP triple quadrupole mass spectrometer (Applied Biosystems/Sciex), coupled to a multiplex LC system that included two 1200-series pumps (Agilent Technologies) and an HTS PAL autosampler (Leap Technologies) equipped with two injection ports and a column selector valve. The pumps were configured for hydrophilic interaction chromatography (HILIC) using a 150 × 2.1 mm Atlantis HILIC column (Waters) and the following mobile phases: mobile phase A consisted of 10 mM ammonium formate with 0.1% formic acid (v/v), and mobile phase B was acetonitrile with 0.1% formic acid (v/v). A 5 μL injection volume was utilized. The multiplex technique facilitated the measurement of 61 metabolite conversions across the two LC systems, with each sample being injected once into each system. The optimized gradient was programmed as follows: each column was eluted isocratically with 5% mobile phase A for one minute, followed by a linear gradient to 60% mobile phase A over 10 minutes.

For MS detection, electrospray ionization (ESI) and multiple reaction monitoring (MRM) scanning in positive ion mode were utilized. Each metabolite was detected by injecting a reference standard before sample analysis. The dwell time for each transition was set at 30 ms, with an iron spray voltage of 4.5 kV and an ion source temperature of 425°C. Internal standard peak areas were monitored for quality control, and individual samples with peak areas deviating from the group mean by more than two standard deviations were re-analyzed.

All sample labeling and LC-MS analysis were conducted in a blinded manner. Sample IDs were randomly coded prior to analysis to ensure that the analysts were unaware of the group assignments.

Quality control (QC) samples were generated by pooling equal volumes of all individual samples and were injected at regular intervals throughout the LC-MS run. Principal component analysis (PCA) of the QC samples showed tight clustering, indicating good instrument stability and reproducibility. The coefficients of variation (CVs) of the internal standards across QC injections were consistently below 15%.

### Metabolite identification

MS raw data were preprocessed using MultiQuant 2.0 (SCIEX), with compounds identified by comparison to purified standards or library entries for frequently occurring unknown entities. All identified metabolites were categorized by superclass, class, and subclass. Detailed classifications and chemical/physical properties can be accessed from the Human Metabolome Database (HMDB; http://www.hmdb.ca/).

### Metabolomic data analysis

The preprocessed metabolite data were imported into MetaboAnalyst 4.0 (http://www.metaboanalyst.ca) for normalization, principal component analysis, partial least squares discriminant analysis, and volcano plotting. Prior to statistical analysis, the dataset was normalized using log transformation and auto-scaling. Principal component analysis (PCA) was performed to assess potential batch effects, and no significant clustering by batch was observed, indicating the absence of systematic batch variation. Differential metabolites (DMs) were identified using Wilcoxon analysis with the criteria of p-value< 0.05 and |logFC| > 0.5. Additionally, MetaboAnalyst was utilized for functional enrichment and metabolic pathway analysis.

### LASSO regression prediction model

After data preprocessing, a dichotomous predictive model was constructed using the LASSO regression method. In this model, differential metabolites served as independent variables, while the diagnosis of diabetes mellitus (0 or 1) was the dependent variable. This approach included variable selection to identify key biomarkers associated with diabetes mellitus. LASSO regression minimizes the sum of squared residuals while incorporating an L1 penalty term on the regression coefficients, defined as follows:


$$\underset{\beta_{0},\beta }{\lim}\frac{1}{2n}\sum_{i=1}^{n}{\left(y_{i}-\beta_{0}-x_{i}^{T}b\right)}^{2}+\lambda \sum_{j=1}^{p}\left|\beta_{j}\right|$$

In the LASSO regression model, nnn represents the number of samples, ppp denotes the number of independent variables, yiy_iyi​ is the value of the dependent variable for the iii-th sample, xix_ixi​ is the vector of independent variables for the iii-th sample, β0\beta_0β0​ is the intercept term, β\betaβ is the vector of regression coefficients, and λ\lambdaλ is the penalty coefficient.

Next, a plot of the regression coefficients against log⁡(λ)\log(\lambda) log(λ) is created. As log⁡(λ)\log(\lambda) log(λ) increases, the regression coefficients gradually decrease towards zero. Using the R package, the regression coefficients corresponding to the optimal alpha value are extracted with the “coef” function, and non-zero regression coefficients are filtered out to identify the key biomarkers.

### T1D rat model

Twenty male Wistar rats (2–3 months old; 300–400 g) were used in this study. The rats were housed in temperature-controlled rooms (24 ± 1°C) with continuous access to food and water. The animal experiments were approved by Hangzhou City University. The rats were divided into two groups of 10. Type 1 diabetes was induced as previously described (14), with rats receiving streptozotocin (STZ, 55 mg/kg, intraperitoneally), while control rats received the same dose of saline via intraperitoneal injection. After STZ injection, weight and blood glucose levels were measured every two days for 4 or 8 weeks. Random blood glucose levels were determined using a portable glucometer (Omron, Shanghai, China) with calibrated test strips. Additionally, peripheral blood levels of glycosylated serum protein (GSP) and insulin were measured at weeks 4 and 8, respectively. Successful diabetes induction in rats was confirmed by measuring fasting blood glucose levels, with values exceeding 250 mg/dL indicating hyperglycemia. Additionally, signs such as polyuria, polydipsia, and weight loss were observed to support the diagnosis.

### Enzyme-linked immunosorbent assay

Serum GSP levels and insulin were determined using specific assay kits. Fasting blood samples were collected from the rats, and the GSP assay kit and insulin ELISA kit (both from Shanghai Sangong Biotechnology, Shanghai, China) were used according to the manufacturer’s instructions. Results were recorded at an OD of 450 nm absorbance using a spectrophotometer (Thermo Fisher Scientific, Waltham, MA, USA).

### Detection of peripheral blood metabolic markers

Peripheral blood samples from the rats were collected and analyzed for three metabolic markers: Hydroxyhexadecanoyl carnitine, Propionylcarnitine, and Valerylcarnitine, using LC-MS. An internal standard solution of D3-Hydroxyhexadecanoyl carnitine, D3-Propionylcarnitine, and D3-Valerylcarnitine at a concentration of 1 μg/mL was added to each serum sample (10 μL). Next, 500 μL of a cold acetonitrile and methanol aqueous solution was added to precipitate proteins, thoroughly mixed, and centrifuged at 3000 rpm for 10 minutes to remove the precipitate. The supernatant was then transferred to a new vial for LC-MS analysis. The chromatographic and mass spectrometry conditions were consistent with those used in previous metabolomics studies. The metabolites were identified by their m/z values as follows: Hydroxyhexadecanoyl carnitine (m/z 402.3 → 85.1), D3-Hydroxyhexadecanoyl carnitine (m/z 405.3 → 88.1), Propionylcarnitine (m/z 218.1 → 85.1), D3-Propionylcarnitine (m/z 221.1 → 88.1), Valerylcarnitine (m/z 246.1 → 85.1), and D3-Valerylcarnitine (m/z 249.1 → 88.1). The relative amounts of the three metabolic markers in each serum sample were calculated using the internal standard method.

### Statistical analysis

To evaluate the validity and reliability of the three metabolic markers as diagnostic tools, ROC curve analysis was conducted using SPSS software version 26.0 (IBM Corporation, Chicago, Illinois, USA). The sensitivity, specificity, accuracy, and AUC (area under the curve) for each marker were calculated and statistically tested, with a significance level set at P< 0.05. LASSO regression was conducted using the glmnet package in R. The optimal λ value (log(λ) = −3.77) was selected based on the minimum mean squared error.

Additionally, all animal experiments were repeated three times, and an unpaired Student’s t-test was employed to compare differences between the two groups. All data are presented as mean ± standard deviation (SD), with P< 0.05 indicating statistical significance.

## Results

### Metabolite profiling

Peripheral blood samples were collected from T1D patients and normal control populations for metabolomic analysis using LC-MS to investigate the metabolic profile of T1D. A total of 157 known metabolites were identified, with 58 detected in ESI- mode and 99 in ESI+ mode. Representative LC-MS spectra of the key metabolites are provided in **Supplementary Figure S1**.

**Figure 1:**
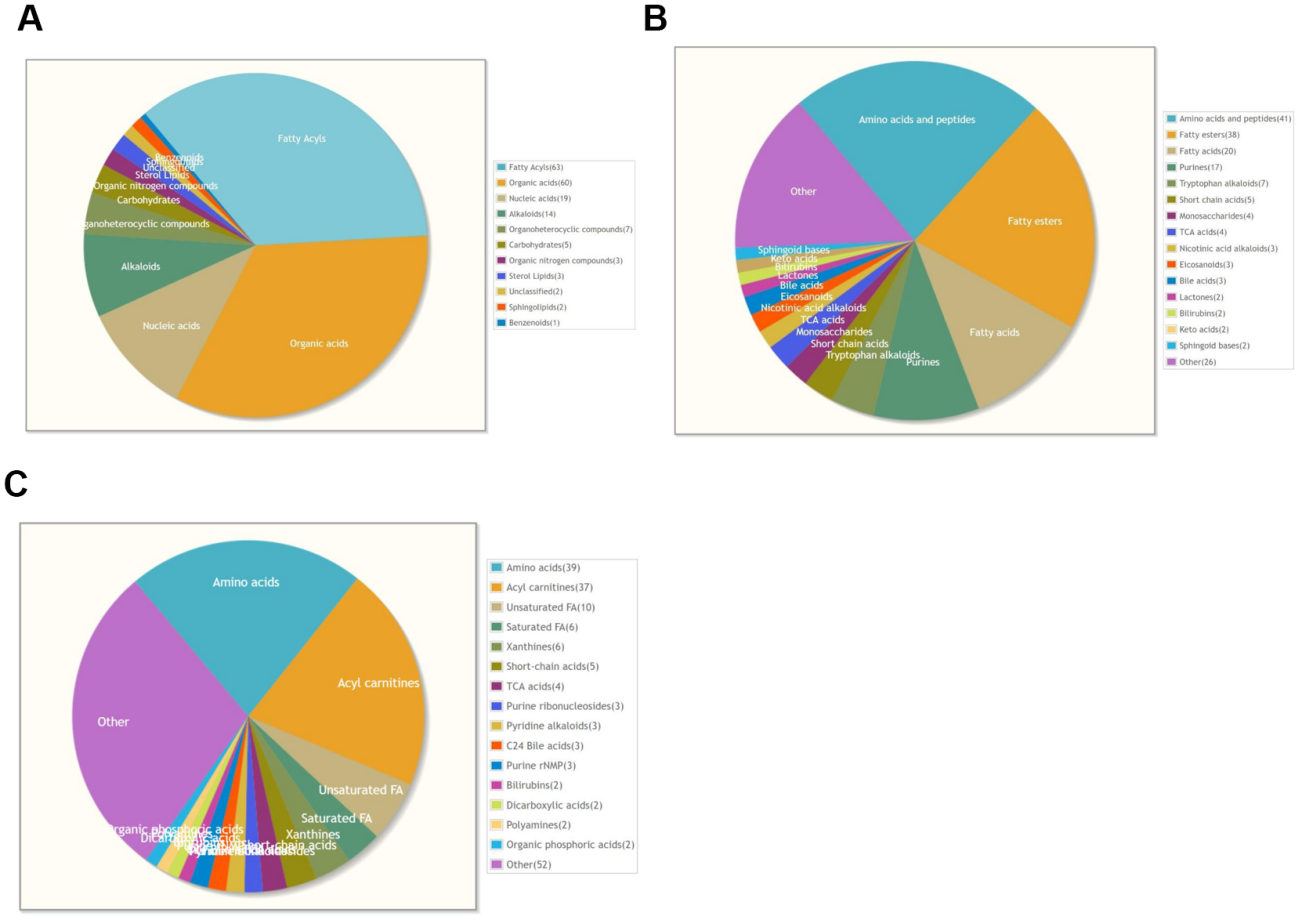
Metabolite profiling. **(A)** Pie chart of metabolite super classes. **(B)** Pie chart of metabolite main classes. **(C)** Pie chart of metabolite sub classes.

Based on metabolite superclasses, the majority of metabolites were categorized into fatty acids (63), organic acids (60), and nucleic acids (19) (**Figure 1A**). Further classification revealed that 41 metabolites fell under the amino acids and peptides class, which constituted the largest group. Additionally, there were significant distributions in fatty esters (38) and fatty acids (20) (**Figure 1B**).

A more detailed analysis showed that approximately 39 metabolites belonged to the amino acids subclass, followed by 37 in the acyl carnitines subclass and 10 in the unsaturated fatty acids subclass (**Figure 1C**).

### Differential metabolites in T1D patients versus normal controls

**Figure 2:**
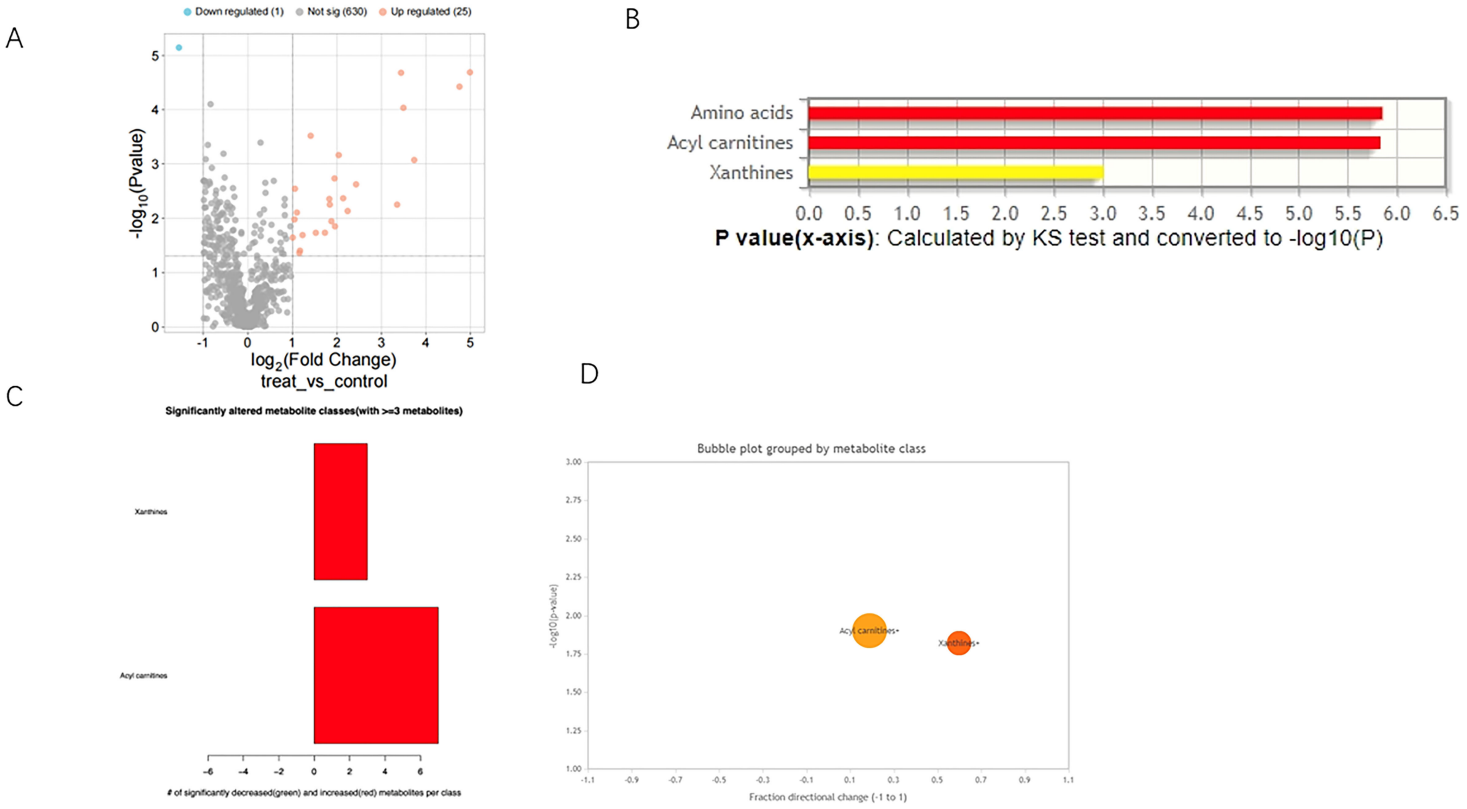
Differential metabolites (DMs) in T1D patients versus normal controls. **(A)** Volcano plot of differential metabolites (DMs) between T1D and healthy controls. **(B)** Class Enrichment of DMs by Kolmorogov-Smirnov test. **(C)** Class Enrichment of DMs displayed in chart. **(D)** Bubble plot of results grouped by metabolite class.

Wilcoxon analysis identified 26 significantly different metabolites (DMs) (P< 0.05, |logFC| > 0.5), of which 25 were up-regulated and one was down-regulated in T1D patient samples (**Figure 2A**). A complete table of all 26 differential metabolites, including fold changes and both raw and FDR-adjusted p-values, was presented in **Supplementary Table S1**. The Kolmogorov-Smirnov test revealed that these metabolites were primarily enriched in the amino acids, acyl carnitines, and xanthines classes (**Figure 2B**). Furthermore, when grouping DMs by metabolite classification, significant differential expression was observed between the acyl carnitines and xanthines classes in T1D patient samples (**Figures 2C, D**).

### Enrichment analysis of differential metabolites

**Figure 3:**
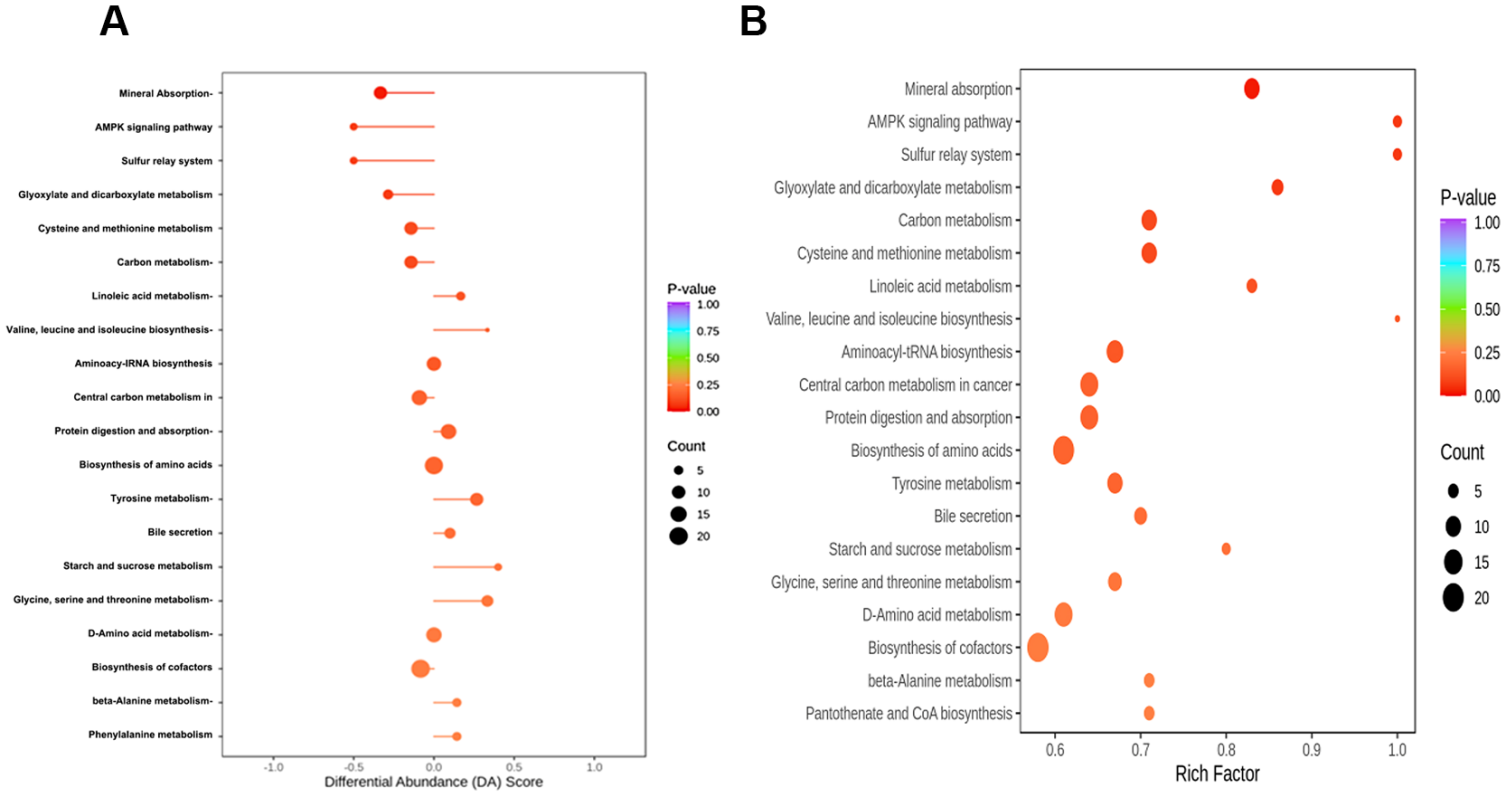
Enrichment analysis of differential metabolites. **(A)** GO analysis shows enrichment in biological processes such as mineral absorption, AMPK signaling, and the sulfur relay system, which are related to energy metabolism and oxidative stress in T1D. **(B)** KEGG pathway analysis reveals enrichment in glyoxylate and dicarboxylate metabolism, carbon metabolism, cysteine and methionine metabolism, and linoleic acid metabolism, indicating disturbances in mitochondrial function, amino acid, and lipid metabolism. Point size reflects the number of metabolites; color indicates statistical significance.

To explore the metabolic profile of T1D, DMs were subjected to pathway enrichment analysis. Gene Ontology (GO) analysis showed that these DMs were primarily associated with biological processes such as mineral absorption, the AMPK signaling pathway, and the sulfur relay system (**Figure 3A**). Notably, the AMPK pathway plays a key role in cellular energy homeostasis and is closely linked to insulin signaling and glucose uptake, suggesting a metabolic adaptation in T1D.

KEGG pathway enrichment analysis revealed that the differential metabolites were mainly involved in glyoxylate and dicarboxylate metabolism, carbon metabolism, cysteine and methionine metabolism, and linoleic acid metabolism (**Figure 3B**). These pathways are associated with mitochondrial energy production, oxidative stress regulation, and lipid metabolism—processes known to be dysregulated in T1D. Together, these findings suggest that metabolic alterations in T1D may reflect disrupted energy balance and redox homeostasis.

### Metabolites associated with the development of T1D were identified through LASSO regression modeling

**Figure 4:**
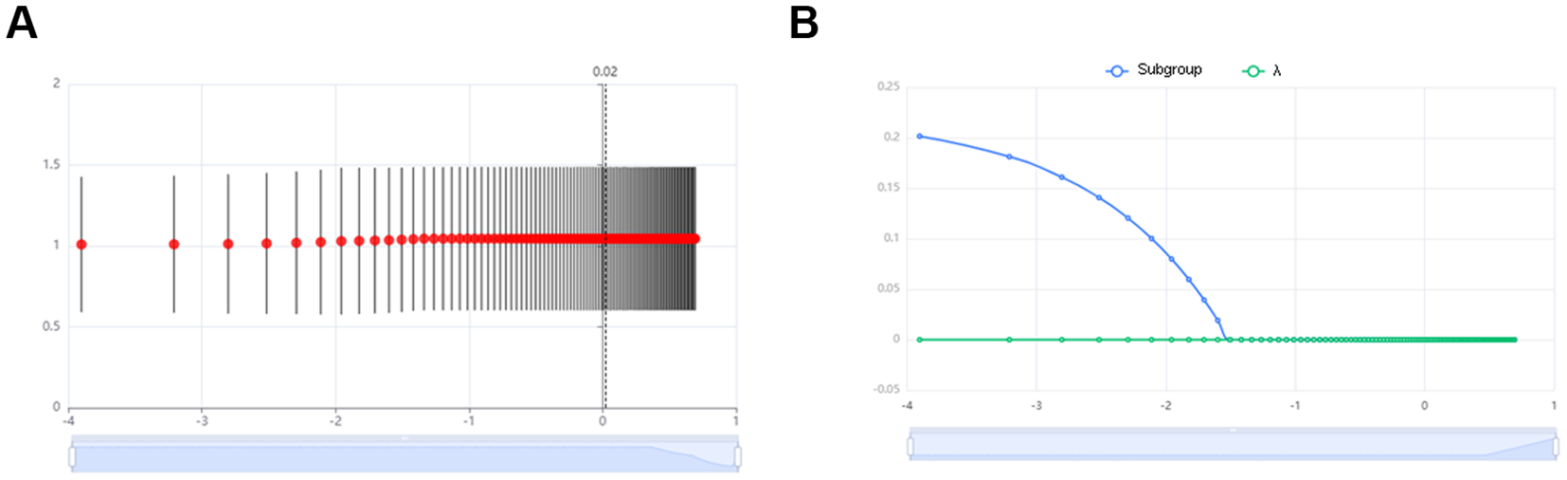
Metabolites associated with the development of T1D were identified through LASSO regression modeling. **(A)** Lasso regression cross-validation plot. Vertical coordinate: model mean square error; Horizontal coordinate: logarithmic value of λ. **(B)** Plot of λ vs. model regression coefficients.

To identify metabolites associated with T1D, a dichotomous predictive model was constructed using LASSO regression. DMs served as independent variables, while diabetes diagnosis (0 or 1) was the dependent variable. The optimal alpha value was found to be -3.77, corresponding to a mean squared error (MSE) of 0.52, which was used to fit the LASSO regression model and determine the final regression coefficients (**Figure 4A**). The plot of regression coefficients against Log(λ) demonstrated that as Log(λ) increased, the coefficients gradually decreased, with some reaching zero (**Figure 4B**). Non-zero coefficients were identified as key metabolites. Ultimately, three key metabolites associated with T1D were identified: Hydroxyhexadecanoyl carnitine (P=0.026, log2 Fold Change=0.751), Propionylcarnitine (P=0.020, log2 Fold Change=0.795), and Valerylcarnitine (P=0.016, log2 Fold Change=0.805). A metabolic pathway map highlighting where Hydroxyhexadecanoyl carnitine and Valerylcarnitine participate in fatty acid β-oxidation and branched-chain amino acid metabolism was shown in **Supplementary Figure S2**. Notably, all three metabolites belonged to the Fatty esters main class and the Acyl carnitines subclass.

### Validation of 3 T1D-related metabolic markers in a diabetic rat model

**Figure 5:**
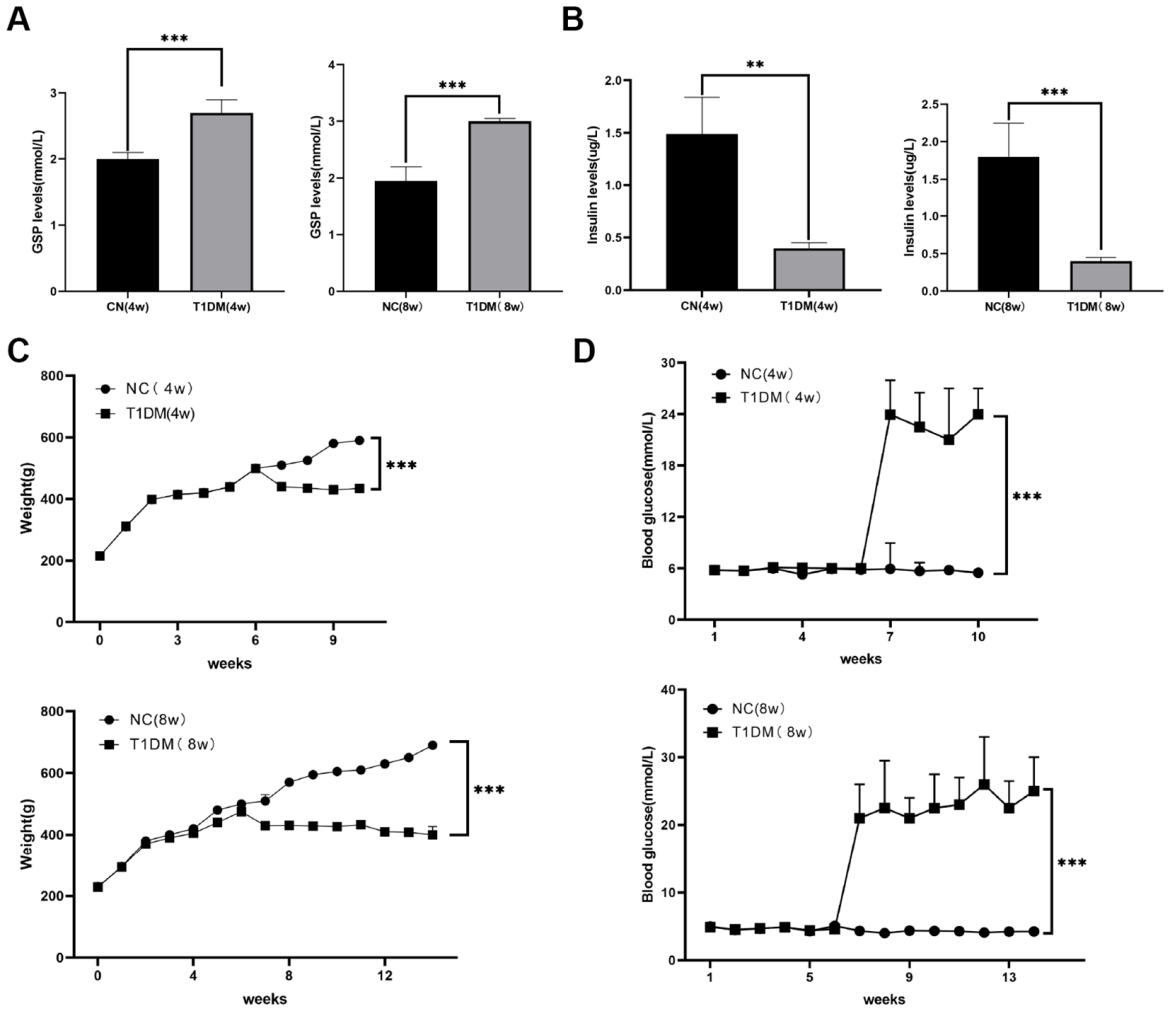
Construction of diabetic rat models. **(A)** Comparison of GSP levels in blood between CN and T1D rat models at 4, 8W. **(B)** Comparison of insulin levels in blood between CN and T1D rat models at 4, 8W. **(C)** Comparison of body weight between CN and T1D rat models at 4, 8W. **(D)** Comparison of blood glucose between CN and T1D rat models at 4, 8W.

A T1D rat model was created to assess the diagnostic potential of metabolic markers through STZ injection. In the T1D group, GSP levels were significantly higher than in the normal control (NC) group at both 4- and 8-weeks post-induction (**Figure 5A**). Additionally, insulin levels were notably reduced in the T1D group compared to NC (**Figure 5B**). Persistently elevated blood glucose levels and decreased body weight confirmed the establishment of the diabetic rat model (**Figures 5C, D**).

**Figure 6:**
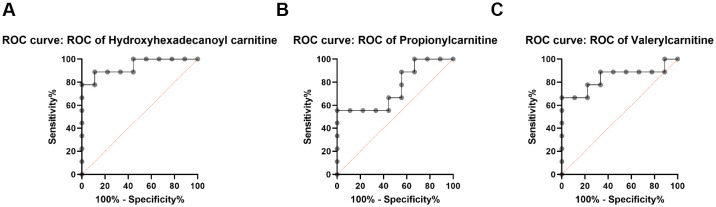
Validation of 3 T1D-related metabolic markers in a diabetic rat model. **(A)** ROC curve of Hydroxyhexadecanoyl carnitine in blood between CN and T1D rat models. **(B)** ROC curve of Propionylcarnitine in blood between CN and T1D rat models. **(C)** ROC curve of Valerylcarnitine in blood between CN and T1D rat models. AUC values are shown with 95% confidence intervals estimated via bootstrapping.

The concentrations of Hydroxyhexadecanoyl carnitine, Propionylcarnitine, and Valerylcarnitine in peripheral blood were analyzed using ROC curve analysis. Results indicated that Hydroxyhexadecanoyl carnitine and Valerylcarnitine were significant diagnostic markers (P< 0.05), with areas under the ROC curve (AUC) of 0.9383 and 0.8395, respectively, both with 95% confidence intervals (CIs) (**Figures 6A–C**). These findings suggest that Hydroxyhexadecanoyl carnitine and Valerylcarnitine could serve as effective indicators for T1D diagnosis.

## Discussion

Metabolomic profiling captures the cumulative effects of both endogenous physiological processes and exogenous influences (15, 16). In this study, we employ advanced untargeted metabolomic analysis to enhance our understanding of T1D disease mechanisms and to identify potential new diagnostic and therapeutic opportunities. The predominance of amino acids, acyl carnitines, and fatty acid subclasses in the metabolomic profile of T1D patients is biologically plausible, as these metabolites are closely linked to insulin regulation, mitochondrial function, and lipid metabolism—key pathways disrupted in type 1 diabetes. Alterations in amino acid metabolism may reflect impaired glucose utilization and increased protein catabolism, while elevated acyl carnitines and fatty acids suggest shifts toward fatty acid oxidation due to insulin deficiency.

In this study, we recruited 50 T1D patients and healthy controls, collecting peripheral blood samples for metabolite analysis using LC-MS technology. We identified a total of 157 known metabolites: 58 in ESI- mode and 99 in ESI+ mode. The metabolites were classified by superclass, class, and subclass, revealing that most belonged to the fatty acid superclass, amino acids and peptides class, and amino acid subclass. Previous studies have also detected a significant presence of fatty acid metabolites in serum samples (17, 18). This is likely due to the distribution of fatty acids in red blood cell (RBC) membranes, which allows for their detection in blood samples (19, 20). Additionally, amino acid metabolites such as arginine-d7, tryptophan-d5, and glutamine-d5 were identified in the blood of diabetic patients (21). These findings align with our results, highlighting the substantial presence of fatty acids and amino acid class compounds in blood samples.

Further analysis using Wilcoxon tests identified 26 significantly DMs in T1D patient samples, with 25 compounds showing up-regulation and one down-regulation. Notable differences were observed in the expression of metabolites from the acylcarnitines and xanthine classes in T1D patient samples.

Acylcarnitines, which are fatty acid metabolites, play crucial roles in various cellular energy metabolic pathways (22). Metabolic abnormalities have been linked to changes in acylcarnitine production and excretion, indicating disruptions in energy metabolism (22, 23). For instance, prior studies have reported elevated serum levels of acylcarnitine metabolites in patients with type 2 diabetes, correlating with an increased risk of developing T2D (24, 25). An increased proportion of Acylcarnitines in hepatic metabolites can lead to enhanced lipid oxidation in the liver (26).

Xanthine, a product of purine metabolism, is converted to uric acid through oxidation by xanthine oxidase (27). Research has shown that xanthine oxidoreductase activity, which catalyzes the oxidation of hypoxanthine to xanthine, is elevated in patients with T2D and correlates with arterial stiffness in these individuals (28). This suggests that higher levels of Xanthines may be present in diabetic patients. Additionally, significant increases in xanthine, hypoxanthine, and AMP levels have been documented in plasma and liver tissues of diabetic rat models, indicating their potential as biomarkers for assessing diabetes progression (29). Together, these findings suggest that Acylcarnitines and xanthine metabolites are present in blood samples from T1D patients and may be closely related to the pathogenesis of the disease.

Emerging algorithms, particularly machine learning methods like LASSO regression, hold great promise for biomarker discovery in disease contexts (30, 31). Many studies have successfully employed machine learning models for disease diagnosis, prognosis, and marker identification. For instance, Jun Liu et al. utilized LASSO regression to identify 24 metabolic markers associated with diabetes, constructing a comprehensive model that integrated fasting glucose, TRF, and metabolic markers to optimize predictive accuracy (12). Similarly, Shi applied a combination of algorithms, including VIP, time series, LASSO, and SVM-RFE, to screen 25 biomarkers related to aging in a training group (32). LASSO regression has also been effective in identifying critical risk factors for prediabetes and diabetes, contributing to the development of early warning models for these conditions (33).

In this study, the LASSO regression method successfully identified key metabolites associated with T1D, including Hydroxyhexadecanoyl carnitine, Propionylcarnitine, and Valerylcarnitine. Notably, all three metabolites are categorized within the fatty ester main class and the acylcarnitine subclass. This aligns with findings from Yue Sun et al., who noted that elevated levels of Valerylcarnitine (C5) are linked to an increased risk of diabetes mellitus (34). Hydroxyhexadecanoyl carnitine (also known as 3-hydroxypalmitoylcarnitine) has been identified at elevated levels in the plasma of individuals with type 2 diabetes, with increased concentrations associated with a heightened risk of diabetes-related cardiovascular complications (35). Furthermore, Propionylcarnitine has demonstrated protective effects against cardiac damage in diabetic patients by alleviating mitochondrial respiratory depression (36). However, it should be noted that Propionylcarnitine, though selected, did not yield strong ROC values, suggesting its individual predictive power may be limited.

These findings suggest that Hydroxyhexadecanoyl carnitine, Propionylcarnitine, and Valerylcarnitine are closely linked to diabetes development. The study’s validation in STZ-induced diabetic rats further confirmed the significance of Hydroxyhexadecanoyl carnitine and Valerylcarnitine as potential diagnostic metabolites for T1D. Ultimately, these metabolites could serve as valuable biomarkers for diagnosing T1D.

The successful validation of only two out of three metabolites in the animal model suggests that while Hydroxyhexadecanoyl carnitine and Valerylcarnitine show strong diagnostic potential for T1D, Propionylcarnitine may lack robustness or biological relevance in this context. This highlights the importance of experimental validation to refine biomarker panels and underscores the need for further studies to confirm the reliability and reproducibility of candidate markers across different models and conditions. Additionally, distinguishing metabolite changes specific to T1D from those caused by STZ-induced metabolic stress requires comparing profiles from STZ-treated non-diabetic controls or dose-variant models. Metabolites consistently altered in both human T1D samples and diabetic rats—but not in STZ-only controls—are more likely to reflect disease-specific changes rather than general toxicity or stress responses.

This study represents a significant advancement in proposing metabolic markers for the diagnosis of T1D; however, it does have limitations. First, the study’s findings need further validation through a larger cohort of clinical samples to confirm the diagnostic validity and risk assessment of the identified metabolic markers. Second, the specific mechanisms by which Hydroxyhexadecanoyl carnitine, Propionylcarnitine, and Valerylcarnitine regulate blood glucose levels remain unclear, highlighting the need for additional experimental evidence to elucidate these pathways. Third, while LASSO regression was used to identify potential metabolic markers, the model selection was based solely on the minimum mean squared error without applying formal k-fold cross-validation or using an external validation set. This lack of validation reduces confidence in the model’s generalizability and predictive performance. Furthermore, confidence intervals for the ROC AUC values were initially omitted, limiting interpretability and assessment of variability in marker performance. Although these intervals have now been added for selected metabolites, comprehensive model uncertainty remains unaddressed. Lastly, the small sample size further restricts the reliability of multivariate modeling and increases the risk of overfitting, which can compromise the reproducibility of the identified markers. Addressing these limitations will be essential for strengthening the reliability and applicability of these metabolic markers in clinical practice.

## Conclusions

In summary, routine metabolomics analysis using LC-MS technology was conducted on peripheral blood samples from pediatric T1D patients and normal controls. A machine learning approach, specifically LASSO regression, was employed to identify key metabolic markers associated with T1D. Of the 26 differentially expressed metabolites, three-Hydroxyhexadecanoyl carnitine, Propionylcarnitine, and Valerylcarnitine-were found to be significantly linked to T1D development. These markers were further validated in a diabetic rat model, where Hydroxyhexadecanoyl carnitine and Valerylcarnitine exhibited superior diagnostic capabilities. These findings suggest that the presence of Hydroxyhexadecanoyl carnitine and Valerylcarnitine in peripheral blood could serve as effective clinical indicators for diagnosing T1D.

## Data Availability

The raw data supporting the conclusions of this article will be made available by the authors, without undue reservation.
