# Supplementary material for: LC-MS-based conventional metabolomics combined with machine learning models to identify metabolic markers for the diagnosis of type I diabetes
Source: Front Endocrinol (Lausanne). 2025 Aug 7;16:1588718. doi: 10.3389/fendo.2025.1588718 (PMC12367486; doi:10.3389/fendo.2025.1588718)
Supplement: Supplementary file 1 [file DataSheet1.docx]

Supplementary materials


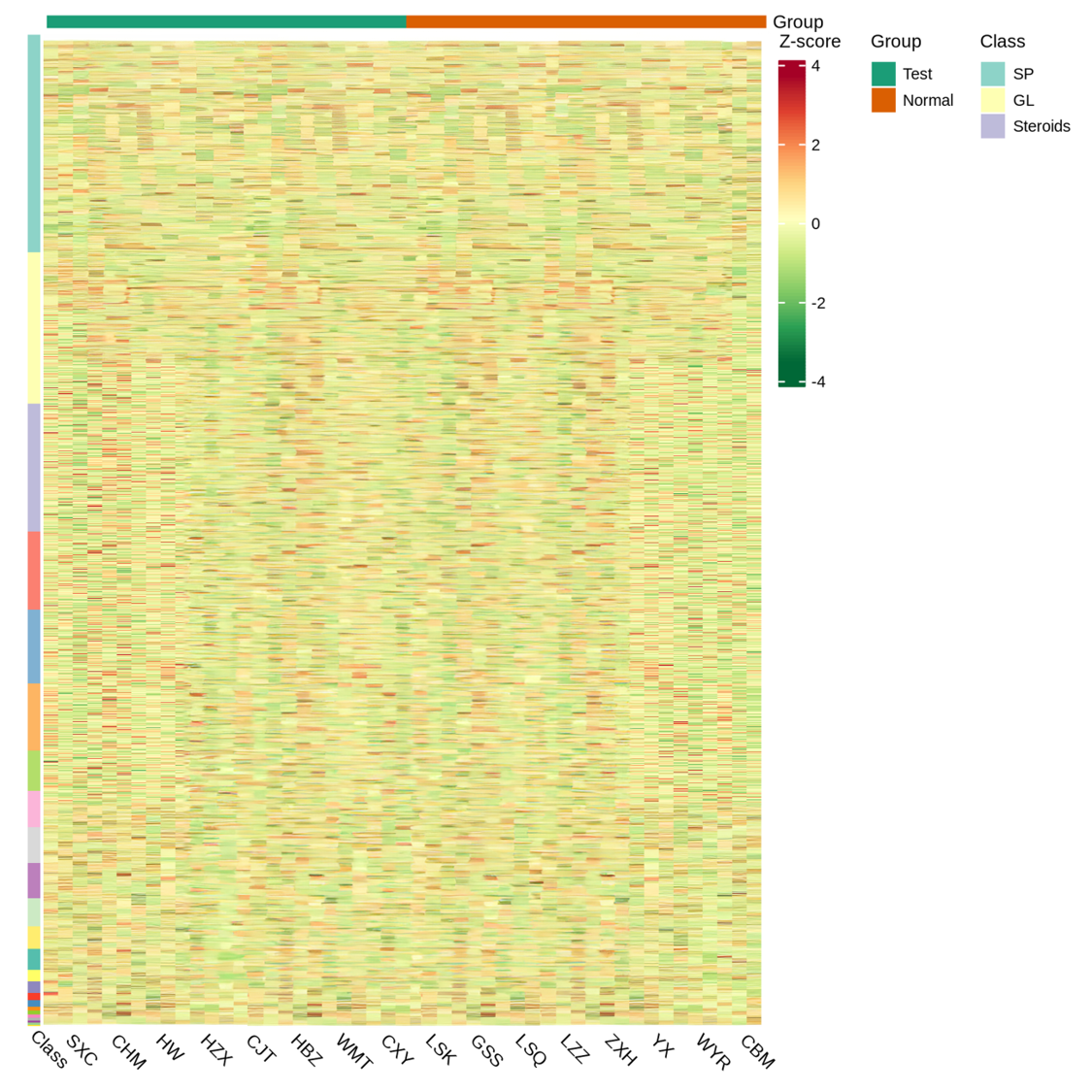


Figure S1. A representative LC-MS spectrum.


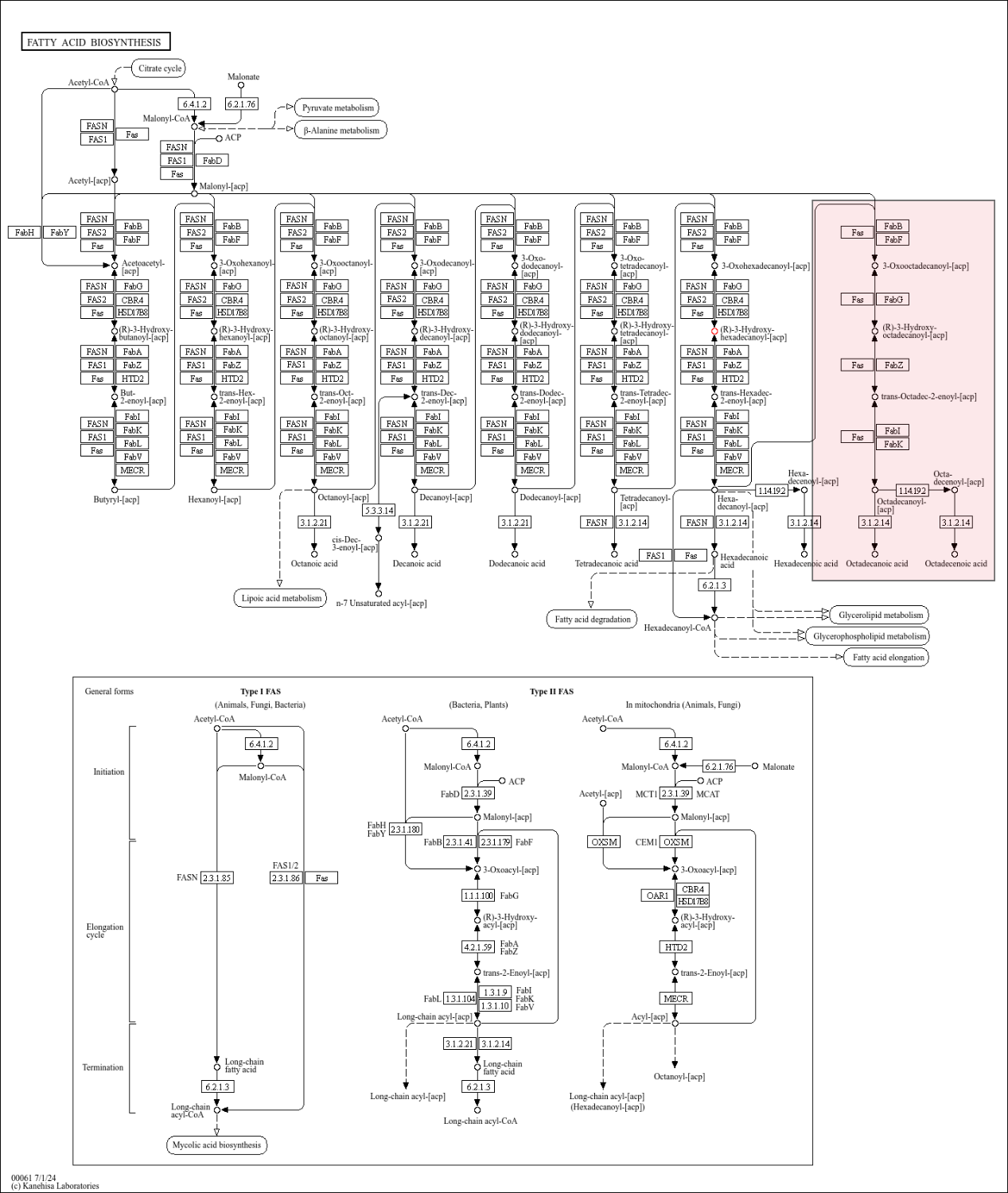


Figure S2. A schematic metabolic pathway map highlighting where Hydroxyhexadecanoyl carnitine and Valerylcarnitine participate in fatty acid β-oxidation and branched-chain amino acid metabolism, respectively.

Table S1. All 26 differential metabolites with p-values and fold changes.

| Name | FC | log2FC | Pvalue | Up/Down |
| --- | --- | --- | --- | --- |
| L-cysteine | 0.343197855697172 | -1.542887556 | 7.15484765311743e-06 | down |
| L-1-Pyrroline-3-hydroxy-5-carboxylate | 31.6759035204121 | 4.985313866134337 | 2.04111477967511e-05 | up |
| L-lysine | 10.8500999333777 | 3.439636425365825 | 2.08164286567336e-05 | up |
| L-leucine | 26.9089342143062 | 4.750013345985176 | 3.75268009438459e-05 | up |
| Ornithine | 11.2522123893805 | 3.4921367842749147 | 9.20033886534214e-05 | up |
| Nicotimide | 2.66033583306162 | 1.411608378850543 | 0.0003017236176524 | up |
| 3-Methyldioxindole | 4.11576846307385 | 2.041161824172899 | 0.0006839418257246 | up |
| Indoxyl | 13.3143959695573 | 3.734915074581896 | 0.0008424883673568 | up |
| Anthranilate | 3.85448042168675 | 1.94653640006881 | 0.0018418088447644 | up |
| Tiglylcarnitine | 5.39228080612776 | 2.430895627650969 | 0.0023686025372998 | up |
| Methylmalonylcarnitine | 2.0845194747876 | 1.0597148507014698 | 0.0028497401030754 | up |
| Valerylcarnitine | 4.41137411139755 | 2.141228114946385 | 0.0042703694073994 | up |
| Propionylcarnitine | 2.77826867320171 | 1.8304733130386504 | 0.0044047831229677 | up |
| Hydroxyhexadecanoylcarnitine | 3.58381098839648 | 1.841494551097044 | 0.0055893926686483 | up |
| Dimethylarginine | 10.2062876830319 | 3.351386307314664 | 0.0055989018825549 | up |
| 4-Acetamidobutanoate | 4.72762645914397 | 2.241116049299265 | 0.0073387646881123 | up |
| Phosphodimethyl-ethanolamine | 2.14700713325752 | 1.1023269844422166 | 0.0078295925001723 | up |
| Kynurenic acid | 2.0695647856203 | 1.0493274113757347 | 0.0105009937065213 | up |
| Creatine | 3.67559568749434 | 1.87797808008534 | 0.011355511114254 | up |
| Taurine | 2.9410685963521 | 1.956851103042981 | 0.0140605195505075 | up |
| 2-Aminooctanoate | 2.66055134925257 | 1.731662336009315 | 0.0184478161145059 | up |
| 2-oxopentanoate | 2.88183802074394 | 1.526989248338224 | 0.0184874014275036 | up |
| Butyrylcarnitine | 2.77745412175939 | 1.2298123226213963 | 0.0203605454079165 | up |
| Phenylacetate (PLA) | 2.013125 | 1.0094367556080903 | 0.0226283105564993 | up |
| Theobromine | 2.25307511122743 | 1.1718954097957357 | 0.039355160469116 | up |
| Theophylline | 2.2312263641275 | 1.1578368864362345 | 0.0433986102204692 | up |
